# Crystal structure of *catena*-poly[[[*trans*-bis(aceto­nitrile-κ*N*)diaquacobalt(II)]-μ-pyrazine-κ^2^ *N*:*N*′] dinitrate]

**DOI:** 10.1107/S2056989016000220

**Published:** 2016-01-13

**Authors:** Chen Liu, Ashley C. Felts, Annaliese E. Thuijs, Aaron Useche, Khalil A. Abboud

**Affiliations:** a Department of Chemistry and Environmental Science, Grenfell Campus, Memorial University of Newfoundland, Corner Brook, NL, A2H 5G4, Canada; b Department of Chemistry, University of Florida, Gainesville, FL, 32611-7200, USA

**Keywords:** crystal structure, one dimensional coordination polymer, cobalt(II) complex, pyrazine ligand, aceto­nitrile ligand

## Abstract

The central structural motif of the title coordination polymer, [Co(NO_3_)_2_(C_4_H_4_N_2_)(CH_3_CN)_2_(H_2_O)_2_]_*n*_, is a chain composed of Co^II^ ions linked by bis-monodentate bridging pyrazine ligands through their N atoms. The Co^II^ ion is located on an inversion center and is additionally coordinated by two O atoms of water mol­ecules and two N atoms of aceto­nitrile mol­ecules. The resultant N_4_O_2_ coordination sphere is distorted octa­hedral. The linear cationic chains extend parallel to the *a* axis and are aligned into layers parallel to the *ac* plane. Nitrate anions are situated in the space between the Co^II^ chains and form O—H⋯O hydrogen bonds with the coordinating water mol­ecules, leading to a three-dimensional network structure. Weak C—H⋯O hydrogen bonds are also present between pyrazine or aceto­nitrile mol­ecules and the nitrate anions.

## Abstract 2

The central structural motif of the title coordination polymer, [Co(C_4_H_4_N_2_)(CH_3_CN)_2_(H_2_O)_2_(NO_3_)_2_]_*n*_, is a chain composed of Co^II^ ions linked by bis-monodentate bridging pyrazine ligands through their N atoms. Nitrate anions are situated in the space between the Co^II^ chains

## Chemical context   

In the design of coordination polymers, the choice of bridging ligands between metal atoms plays an important role in the formation of the final structure and the resulting properties. During our investigations of the preparation conditions and magnetic properties of compounds with ladder-like structures, we have used pyrazine as a bis-monodentate bridging ligand to link paramagnetic metal cations. From the point of view of mediating magnetic inter­actions, the pyrazine mol­ecule offers some advantages compared to other bidentate bridging ligands such as 4,4′-bi­pyridine. In some of the structures with the latter ligand, the two pyridine rings are not co-planar and therefore can magnetically isolate metal atoms (Losier & Zaworotko, 1996 ▸; Ruan *et al.*, 2009 ▸; Seidel *et al.*, 2011 ▸; Lehleh *et al.*, 2013 ▸).
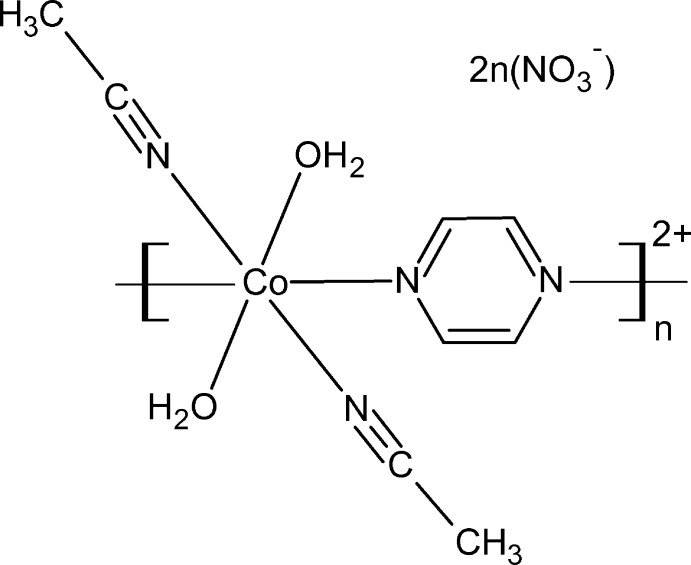


We herein report the preparation and structure of a pyrazine-bridged chain structure obtained by reacting pyrazine and cobalt(II) nitrate hexa­hydrate using aceto­nitrile as the solvent.

## Structural commentary   

**Figure 1:**
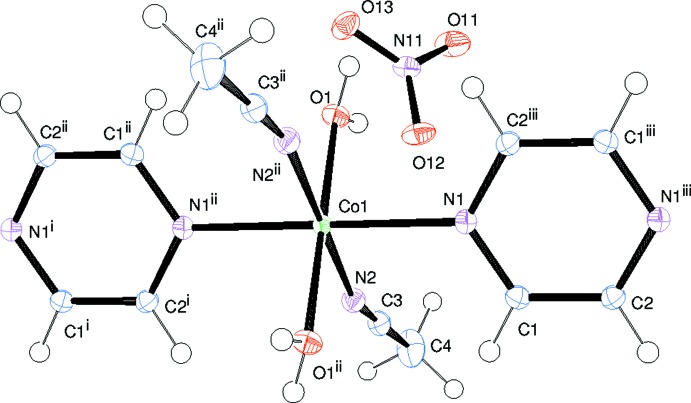
A fragment of the one-dimensional chain structure of the title compound with displacement ellipsoids drawn at the 50% probability level. [Symmetry codes: (i) 1 + *x*, *y*, *z*; (ii) −3 − *x*, 1 − *y*, −*z*; (iii) −4 − *x*, 1 − *y*, −*z*.]

The asymmetric unit of the title compound, [Co(C_4_H_4_N_2_)(CH_3_CN)_2_(H_2_O)_2_(NO_3_)_2_]_*n*_, contains one Co^II^ cation located on an inversion center, one water mol­ecule, one aceto­nitrile mol­ecule, one nitrate anion, and one half of a pyrazine mol­ecule, the latter being completed by inversion symmetry. The Co^II^ cation exhibits an N_4_O_2_ coordination set defined by two O atoms [O1, O1^ii^; symmetry code: (ii) −3 − *x*, 1 − *y*, −*z*] of two coordinating water mol­ecules, two N atoms (N2, N2^ii^) of two coordinating aceto­nitrile mol­ecules, and two nitro­gen atoms (N1, N1^ii^) of two bridging pyrazine mol­ecules (Fig. 1 ▸). The two Co—O_water_ bonds have a length of 2.0315 (8) Å, considerably shorter than the two Co—N_aceto­nitrile_ bonds of 2.1263 (9) Å, and the two Co—N_pyrazine_ bonds of 2.1493 (10) Å. The resulting coordination sphere is compressed octa­hedral with all bond lengths in good agreement with similar structures (Choudhury *et al.*, 2002 ▸; Holman *et al.*, 2005 ▸; Aşkin *et al.*, 2015 ▸). In contrast to the N_2_O_4_ coordination spheres observed more frequently in the structures of other Co-containing compounds (Choudhury *et al.*, 2002 ▸; Holman *et al.*, 2005 ▸; Hyun *et al.*, 2011 ▸; Aşkin *et al.*, 2015 ▸), the title structure exhibits an N_4_O_2_ coordination sphere due to the inclusion of the solvent aceto­nitrile mol­ecules in the coordination sphere of Co^II^. The bridging bis-monodentate pyrazine mol­ecules link the Co^II^ ions, forming linear chains extending parallel to the *a* axis. The distance between two symmetry-related Co^II^ ions within a chain (symmetry code: 1 + *x*, *y*, *z*) is 7.0798 (3) Å, in good agreement with those reported for similar structures (Choudhury *et al.*, 2002 ▸; Holman *et al.*, 2005 ▸; Aşkin *et al.*, 2015 ▸).

## Supra­molecular features   

**Figure 2:**
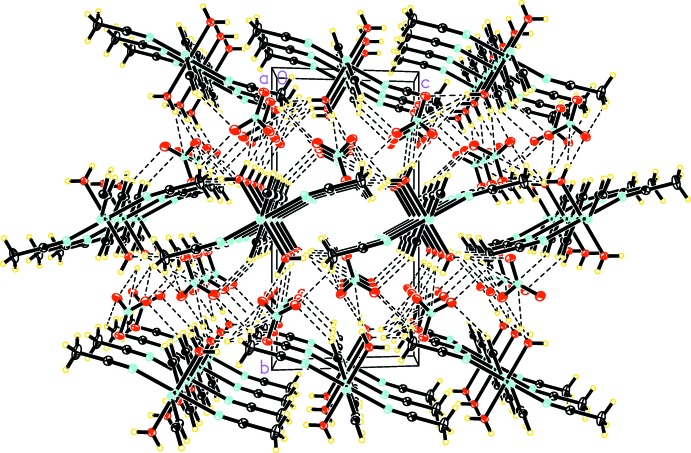
Crystal packing of the title compound, showing hydrogen bonds as dashed lines.

In the crystal, the cationic chains are arranged to form sheets parallel to the *ac* plane, and neighboring sheets are related by a glide plane. Nitrate ions are sandwiched in the space between the sheets and form columns parallel to the *a* axis. Each Co^II^ chain is surrounded by six columns of nitrate ions that are related by the inversion centers located along the cationic chains. Each cationic chain is further surrounded by six other chains. This structural motif with alternating layers has been observed in similar structures (Choudhury *et al.*, 2002 ▸; Yang *et al.*, 2003 ▸; Holman *et al.*, 2005 ▸; Aşkin *et al.*, 2015 ▸). Co^II^ chains in neighboring sheets inter­act through nitrate ions by forming O—H⋯O hydrogen bonds where the donor O—H groups are provided by the coordinating water mol­ecules and the acceptor oxygen provided by the nitrate ions. One of those hydrogen bonds is bifurcated. For numerical values and symmetry operators, see Table 1 ▸. Weak C—H⋯O hydrogen bonds are also present between the C—H groups of bridging pyrazine and coordinating aceto­nitrile mol­ecules, and the oxygen atoms of nitrate ions, linking Co^II^ chains both within the same sheet and to adjacent sheets (Table 1 ▸, Fig. 2 ▸).

## Synthesis and crystallization   

The title compound was obtained by a slow diffusion method in an U-shaped glass tube. The tube was first partially filled with aceto­nitrile. An aceto­nitrile solution of 0.333 mmol (97.0 mg) of Co(NO_3_)_2_·6H_2_O was then placed in one arm of the tube. Another aceto­nitrile solution of 0.500 mmol (40.0 mg) of pyrazine was placed in the other arm of the tube. The slow diffusion of the two solutions in the tube produced pink needle–shaped crystals within one day. The crystals were collected by filtration and washed with fresh aceto­nitrile and kept under inert atmosphere (yield 31.5%). Selected IR bands (KBr, cm^−1^): 3273 (O—H), 2283 (C N), 1633, 1413, 1384 (N=O), 479 (bridging pyrazine).

## Refinement details   

Crystal data, data collection and structure refinement details are summarized in Table 2 ▸. C-bound H atoms were calculated in geometrically idealized positions and refined riding on their parent atoms, with *U*_iso_(H) = 1.2*U*_eq_(C) (aromatic) and 1.5*U*_eq_(C) (meth­yl), and with C—H = 0.95 Å (aromatic) and 0.98 Å (meth­yl). The methyl H atoms were allowed to rotate around the corresponding C—C bond. H atoms bound to water mol­ecules were found in a difference map and were freely refined.

## Supplementary Material

Crystal structure: contains datablock(s) I. DOI: 10.1107/S2056989016000220/wm5260sup1.cif

Structure factors: contains datablock(s) I. DOI: 10.1107/S2056989016000220/wm5260Isup2.hkl

CCDC reference: 1445438

Additional supporting information:  crystallographic information; 3D view; checkCIF report

## Figures and Tables

**Table 1:** Hydrogen-bond geometry (Å, °)

*D*—H⋯*A*	*D*—H	H⋯*A*	*D*⋯*A*	*D*—H⋯*A*
O1—H1*Y*⋯O13^i^	0.83 (2)	1.85 (2)	2.6819 (12)	173.5 (18)
O1—H1*X*⋯O12^ii^	0.80 (2)	1.99 (2)	2.7869 (12)	174.1 (19)
O1—H1*X*⋯O13^ii^	0.80 (2)	2.562 (19)	3.0912 (12)	125.3 (17)
C1—H1*A*⋯O11^iii^	0.95	2.54	3.1572 (14)	123
C2—H2*A*⋯O13^iv^	0.95	2.59	3.4644 (14)	153
C4—H4*A*⋯O13^v^	0.98	2.49	3.2785 (17)	138
C4—H4*B*⋯O11^vi^	0.98	2.49	3.2823 (17)	138

Symmetry codes: (i) 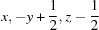; (ii) 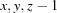; (iii) 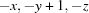; (iv) 
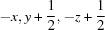
; (v) 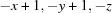; (vi) 
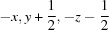
.

**Table 2:** Experimental details

Crystal data	
Chemical formula	[Co(NO_3_)_2_(C_4_H_4_N_2_)(C_2_H_3_N)_2_(H_2_O)_2_]
*M*_r_	381.18
Crystal system, space group	Monoclinic, *P*2_1_/*c*
Temperature (K)	100
*a*, *b*, *c* (Å)	7.0798 (3), 15.0376 (6), 7.9329 (3)
β (°)	110.8803 (6)
*V* (Å^3^)	789.10 (5)
*Z*	2
Radiation type	Mo *K*α
μ (mm^−1^)	1.14
Crystal size (mm)	0.29 × 0.11 × 0.08

Data collection	
Diffractometer	Bruker APEXII DUO CCD
Absorption correction	Analytical based on measured indexed crystal faces using *SHELXTL2014* (Sheldrick, 2015*b* ▸)
*T*_min_, *T*_max_	0.735, 0.904
No. of measured, independent and observed [*I* > 2σ(*I*)] reflections	21292, 1811, 1687
*R*_int_	0.022
(sin θ/λ)_max_ (Å^−1^)	0.650

Refinement	
*R*[*F* ^2^ > 2σ(*F* ^2^)], *wR*(*F* ^2^), *S*	0.019, 0.055, 1.07
No. of reflections	1811
No. of parameters	115
H-atom treatment	H atoms treated by a mixture of independent and constrained refinement
Δρ_max_, Δρ_min_ (e Å^−3^)	0.34, −0.31

Computer programs: *APEX2* and *SAINT* (Bruker, 2014 ▸), *SHELXLT* (Sheldrick, 2015*a* ▸), *SHELXL2014*/7 (Sheldrick, 2015*b* ▸), *XP* in *SHELXTL-Plus* (Sheldrick, 2008 ▸), *ORTEP-3 for Windows* (Farrugia, 2012 ▸) and *publCIF* (Westrip, 2010 ▸).

## supplementary crystallographic information

### Crystal data

**Table:**

[Co(NO_3_)_2_(C_4_H_4_N_2_)(C_2_H_3_N)_2_(H_2_O)_2_]	*F*(000) = 390
*M**_r_* = 381.18	*D*_x_ = 1.604 Mg m^−^^3^
Monoclinic, *P*2_1_/*c*	Mo *K*α radiation, λ = 0.71073 Å
*a* = 7.0798 (3) Å	Cell parameters from 9896 reflections
*b* = 15.0376 (6) Å	θ = 2.0–28.0°
*c* = 7.9329 (3) Å	µ = 1.14 mm^−^^1^
β = 110.8803 (6)°	*T* = 100 K
*V* = 789.10 (5) Å^3^	Needle, pink
*Z* = 2	0.29 × 0.11 × 0.08 mm

### Data collection

**Table:**

Bruker APEXII DUO CCD diffractometer	1687 reflections with *I* > 2σ(*I*)
Radiation source: fine-focus sealed tube	*R*_int_ = 0.022
φ– and ω–scans	θ_max_ = 27.5°, θ_min_ = 2.7°
Absorption correction: analytical based on measured indexed crystal faces using *SHELXTL2014* (Sheldrick, 2015*b*)	*h* = −9→9
*T*_min_ = 0.735, *T*_max_ = 0.904	*k* = −19→19
21292 measured reflections	*l* = −10→10
1811 independent reflections	

### Refinement

**Table:**

Refinement on *F*^2^	0 restraints
Least-squares matrix: full	Hydrogen site location: mixed
*R*[*F*^2^ > 2σ(*F*^2^)] = 0.019	H atoms treated by a mixture of independent and constrained refinement
*wR*(*F*^2^) = 0.055	*w* = 1/[σ^2^(*F*_o_^2^) + (0.0316*P*)^2^ + 0.2947*P*] where *P* = (*F*_o_^2^ + 2*F*_c_^2^)/3
*S* = 1.07	(Δ/σ)_max_ < 0.001
1811 reflections	Δρ_max_ = 0.34 e Å^−^^3^
115 parameters	Δρ_min_ = −0.31 e Å^−^^3^

### Special details

**Table:**

Geometry. All e.s.d.'s (except the e.s.d. in the dihedral angle between two l.s. planes) are estimated using the full covariance matrix. The cell e.s.d.'s are taken into account individually in the estimation of e.s.d.'s in distances, angles and torsion angles; correlations between e.s.d.'s in cell parameters are only used when they are defined by crystal symmetry. An approximate (isotropic) treatment of cell e.s.d.'s is used for estimating e.s.d.'s involving l.s. planes.

### Fractional atomic coordinates and isotropic or equivalent isotropic displacement parameters (Å^2^)

**Table:**

	*x*	*y*	*z*	*U*_iso_*/*U*_eq_	
Co1	0.5000	0.5000	0.0000	0.00977 (8)	
O1	0.45432 (12)	0.38331 (6)	−0.13782 (12)	0.01522 (17)	
H1Y	0.431 (3)	0.3365 (13)	−0.092 (2)	0.035 (5)*	
H1X	0.391 (3)	0.3842 (13)	−0.243 (3)	0.036 (5)*	
N1	0.19570 (15)	0.49931 (6)	−0.00181 (13)	0.01163 (19)	
N2	0.39794 (14)	0.56807 (6)	−0.25144 (13)	0.01470 (19)	
C1	0.07744 (16)	0.57128 (7)	−0.05354 (15)	0.0134 (2)	
H1A	0.1283	0.6228	−0.0923	0.016*	
C2	−0.11795 (16)	0.57209 (7)	−0.05167 (14)	0.0131 (2)	
H2A	−0.1981	0.6242	−0.0889	0.016*	
C3	0.31802 (17)	0.58923 (8)	−0.39744 (16)	0.0158 (2)	
C4	0.2144 (2)	0.61342 (10)	−0.58544 (17)	0.0278 (3)	
H4A	0.3122	0.6380	−0.6346	0.042*	
H4B	0.1106	0.6580	−0.5939	0.042*	
H4C	0.1508	0.5605	−0.6545	0.042*	
N11	0.23020 (15)	0.30295 (7)	0.44860 (13)	0.0172 (2)	
O11	0.09637 (15)	0.26787 (6)	0.31960 (13)	0.0295 (2)	
O12	0.21017 (14)	0.38082 (6)	0.49826 (12)	0.0228 (2)	
O13	0.39023 (13)	0.26094 (6)	0.53329 (12)	0.0230 (2)	

### Atomic displacement parameters (Å^2^)

**Table:**

	*U*^11^	*U*^22^	*U*^33^	*U*^12^	*U*^13^	*U*^23^
Co1	0.00857 (12)	0.01064 (12)	0.01028 (12)	−0.00011 (7)	0.00357 (8)	0.00023 (7)
O1	0.0187 (4)	0.0130 (4)	0.0136 (4)	−0.0023 (3)	0.0053 (3)	−0.0011 (3)
N1	0.0107 (4)	0.0130 (5)	0.0112 (4)	0.0000 (3)	0.0038 (3)	−0.0005 (3)
N2	0.0142 (4)	0.0151 (5)	0.0151 (5)	0.0002 (4)	0.0056 (4)	0.0010 (4)
C1	0.0135 (5)	0.0125 (5)	0.0144 (5)	−0.0006 (4)	0.0051 (4)	0.0011 (4)
C2	0.0128 (5)	0.0127 (5)	0.0138 (5)	0.0012 (4)	0.0045 (4)	0.0013 (4)
C3	0.0144 (5)	0.0164 (5)	0.0178 (6)	−0.0005 (4)	0.0072 (4)	0.0008 (4)
C4	0.0215 (6)	0.0426 (8)	0.0162 (6)	0.0002 (6)	0.0029 (5)	0.0093 (5)
N11	0.0222 (5)	0.0137 (5)	0.0149 (4)	−0.0015 (4)	0.0057 (4)	0.0004 (4)
O11	0.0319 (5)	0.0190 (5)	0.0236 (5)	−0.0026 (4)	−0.0073 (4)	−0.0018 (4)
O12	0.0315 (5)	0.0134 (4)	0.0213 (4)	0.0030 (4)	0.0067 (4)	−0.0024 (3)
O13	0.0207 (4)	0.0198 (4)	0.0232 (5)	0.0041 (3)	0.0012 (3)	−0.0054 (3)

### Geometric parameters (Å, º)

**Table:**

Co1—O1	2.0315 (8)	C1—C2	1.3888 (15)
Co1—O1^i^	2.0315 (8)	C1—H1A	0.9500
Co1—N2^i^	2.1263 (9)	C2—N1^ii^	1.3425 (14)
Co1—N2	2.1263 (9)	C2—H2A	0.9500
Co1—N1^i^	2.1493 (10)	C3—C4	1.4542 (16)
Co1—N1	2.1493 (10)	C4—H4A	0.9800
O1—H1Y	0.83 (2)	C4—H4B	0.9800
O1—H1X	0.80 (2)	C4—H4C	0.9800
N1—C1	1.3401 (14)	N11—O11	1.2378 (13)
N1—C2^ii^	1.3425 (14)	N11—O12	1.2595 (13)
N2—C3	1.1383 (15)	N11—O13	1.2616 (13)
			
O1—Co1—O1^i^	180.0	C1—N1—Co1	120.89 (7)
O1—Co1—N2^i^	91.42 (4)	C2^ii^—N1—Co1	121.65 (7)
O1^i^—Co1—N2^i^	88.58 (4)	C3—N2—Co1	165.59 (9)
O1—Co1—N2	88.58 (4)	N1—C1—C2	121.32 (10)
O1^i^—Co1—N2	91.42 (4)	N1—C1—H1A	119.3
N2^i^—Co1—N2	180.0	C2—C1—H1A	119.3
O1—Co1—N1^i^	88.55 (3)	N1^ii^—C2—C1	121.23 (10)
O1^i^—Co1—N1^i^	91.45 (3)	N1^ii^—C2—H2A	119.4
N2^i^—Co1—N1^i^	89.51 (4)	C1—C2—H2A	119.4
N2—Co1—N1^i^	90.49 (4)	N2—C3—C4	178.24 (13)
O1—Co1—N1	91.45 (3)	C3—C4—H4A	109.5
O1^i^—Co1—N1	88.55 (3)	C3—C4—H4B	109.5
N2^i^—Co1—N1	90.49 (4)	H4A—C4—H4B	109.5
N2—Co1—N1	89.51 (4)	C3—C4—H4C	109.5
N1^i^—Co1—N1	180.0	H4A—C4—H4C	109.5
Co1—O1—H1Y	121.0 (12)	H4B—C4—H4C	109.5
Co1—O1—H1X	118.3 (14)	O11—N11—O12	121.09 (10)
H1Y—O1—H1X	110.2 (18)	O11—N11—O13	120.29 (10)
C1—N1—C2^ii^	117.45 (10)	O12—N11—O13	118.61 (10)

Symmetry codes: (i) −*x*+1, −*y*+1, −*z*; (ii) −*x*, −*y*+1, −*z*.

### Hydrogen-bond geometry (Å, º)

**Table:**

*D*—H···*A*	*D*—H	H···*A*	*D*···*A*	*D*—H···*A*
O1—H1*Y*···O13^iii^	0.83 (2)	1.85 (2)	2.6819 (12)	173.5 (18)
O1—H1*X*···O12^iv^	0.80 (2)	1.99 (2)	2.7869 (12)	174.1 (19)
O1—H1*X*···O13^iv^	0.80 (2)	2.562 (19)	3.0912 (12)	125.3 (17)
C1—H1*A*···O11^ii^	0.95	2.54	3.1572 (14)	123
C2—H2*A*···O13^v^	0.95	2.59	3.4644 (14)	153
C4—H4*A*···O13^i^	0.98	2.49	3.2785 (17)	138
C4—H4*B*···O11^vi^	0.98	2.49	3.2823 (17)	138

Symmetry codes: (i) −*x*+1, −*y*+1, −*z*; (ii) −*x*, −*y*+1, −*z*; (iii) *x*, −*y*+1/2, *z*−1/2; (iv) *x*, *y*, *z*−1; (v) −*x*, *y*+1/2, −*z*+1/2; (vi) −*x*, *y*+1/2, −*z*−1/2.
